# Iodine Injections in Leucorrhœa

**Published:** 1854-11

**Authors:** 


Iodine Injections in Leucorrhœa. By Dr. Russell.—It is not my design, in this communication, to enter into any theoretical inquiries respecting the nature of leucorrhœa, or the *modus operandi* of the remedy I propose for its cure; my object being simply to give the results of my experience in relation to iodine as a remedial agent in this obstinate, and in many cases intractable disease; and in doing which, I shall record facts, well assured that one well-attested fact contributes more to the advancement of the science of medicine than three-fourths of the theories to which the press has ever given publicity.

I consider this disease to consist in inflammation of the vagina or the internal cavity of the uterus, or of both; and in the majority of those cases which have continued for a number of years and resisted the ordinary modes of treatment, ulceration to a greater or less extent will generally be found to exist. For this condition, I have found no remedy equal to iodine; and, in illustration of its effects in my hands, I will briefly detail several cases from a number that have come under my observation:—

Case 1.—In July, 1848, I was requested, by Mr. W——, to visit Grace, a favorite mulatto servant, aged forty eight years, who he informed me, had leucorrhœa of twelve years' standing; had been under the care of a number of physicians during this period, and had been subjected to a great variety of treatment, but without avail. I found her confined to bed, very much debilitated and emaciated, face cadaverous, pulse quick and feeble, skin cool, urine scanty, severe pains in the lumbar and pelvic regions, and œdema of the lower extremities. The vaginal discharge, often escaping by gushes, was excessive, and her general health had become seriously involved from the effects of this long-continued drain to the constitution. Upon examination per vaginam, the vagina, os, and cervix uteri were found to be in a sub inflamed condition, and denuded of epithelium. The os was partially everted, and when deprived of its adherent mucus, presented a vermilion color. The external cervix was enlarged, indurated, and ulcerated. The body of the uterus was sensibly enlarged, and descended within two inches of the os externum. The secretions from these several parts varied essentially in character, and when discharged externally resembled somewhat, in quantity, consistence, and color, the yolk of an egg intermixed with purulent and sanguinolent matter, and all blended in a thick, opaque, tenacious plasma.

Ascertaining that she had never used iodine in any form, and believing it would afford her relief, I ordered the aqueous solution of the following strength to be thrown up the vagina twice daily, and retained several minutes, the parts being previously well syringed with warm water and castile soap, and the patient placed in a horizontal position, with the hips elevated:

℞. Iodini, gr. i; potass. iodid., gr. ii; aquæ pluvialis ℥i. M.

As this solution ceased to create any sensation of warmth or excitement in the parts, it was gradually increased to treble its strength. The muriated tincture of iron, in the proportion of twenty drops three times daily, was given as a tonic.

Under this treatment, with a nourishing diet, she soon began to improve; the irritated condition of the parts gradually subsided, the muco-purulent discharges by degrees ceased, the cervical ulcers regularly healed, and at the end of three months from its commencement, she had regained her health and strength to such an extent as to enable her to resume her occupation as cook to the family. I may add, that her mammæ, which was very small and flaccid, became full and enlarged while under the influence of this medicine, and for several weeks secreted rather copiously a brownish watery fluid. This secretion being tested with starch, produced the characteristic blue color, showing that the iodine was absorbed.

Case 2.—August, 1848. Mrs. W—— consulted me; she was aged nineteen years; small and delicate figure; had been married four years, and dates the commencement of her present "weakness" to an abortion which occurred about six months subsequent to her marriage. Prior to marriage was remarkably healthy and active. At the time I saw her, she was anemic and emaciated, countenance chlorotic, eyes sunken, pulse feeble, menstruation painful. and either scanty or profuse. The vaginal discharge was constant and copious, muco-purulent, slightly streaked with blood, and very offensive. An examination, with the speculum, revealed an irritated condition of the vagina, with relaxation and loss of its natural rugæ, and accompanied by a partial displacement of the uterus. The cervix was enlarged and indurated, with several ulcers upon its external surface. She had never submitted to medical advice, contenting herself with the use of some simple domestic remedies. Correcting the torpid condition of her liver by means of the usual remedies, I prescribed the aqueous solution of iodine and the muriated tincture of iron as above, recommending a nourishing diet, with free exercise in the open air.

She gradually improved under this plan of treatment, and in a few months her general health was re-established. She has, I understand, continued well ever since.

---

Case 3.—October, 1850. Mrs. G——, aged about twenty-seven years, large frame, mixed temperament, five years married, but has never been pregnant. Says that she was never "sick" previous to marriage, but subsequent thereto has always been in "delicate health." Has enlargement and induration of the liver and spleen, sequelæ of the intermittent fever, menstruation irregular and profuse. She was sallow and exsanguined, and exhibited in a great degree, that long train of symptoms consequent upon an obstinate and protracted leucorrhœa. The vaginal discharge was constant, but variable in quantity and quality. Occasionally it was thin and acrimonious, often viscid and scanty, but usually purulent or muco-purulent, and excessive. The cervix was soft and tender to the touch, and when seen by the speculum was found enlarged and presenting a dark greyish appearance. The os was patulous, with tumefied edges, and of a reddish tint. A slight abrasion was found on the posterior lip.

Astringent vaginal injections—as the nitras argenti, acetas plumbi, &c.,—were advised, and to relieve the enlarged and indurated condition of the liver and spleen, I prescribed the following:

℞. Prot. iod. mer. ℈i; pulv. aloes ℈iss; ext. hyoscyami ʒi. M. Div. in pilulæ xxiv. One pill to be taken every night; at the same time five drops of nitro-muriatic acid in a wine-glass full of the infusion of gentian, three times daily, was administered.

Under this treatment, the visceral derangements totally disappeared in about eight weeks, and her general health was greatly restored.

The leucorrhœa still continuing (no benefit having resulted from the use of astringent injections), and no amelioration in the condition of the parts being found on a second vaginal examination which was now made, I ordered the aqueous solution of iodine and the muriated tincture of iron as above recommended. In six weeks after using these remedies, she declared herself well; shortly afterwards became pregnant, and was in time delivered of a fine healthy child.

Other cases could be adduced to prove the remedial powers of iodine as a local remedy in leucorrhœa, but as they are somewhat similar to the above in all essential particulars, it is unnecessary to introduce them here. Regarding the disease as being essentially a local one, our mode of treatment is principally local, and applied by means of a proper syringe to the parts affected. The preparation should at first be made weak, and gradually strengthened as the parts become accustomed to its application. The mildest preparation is frequently disagreeable, and sometimes painful, but these sensations are only momentary.

We have used it varying in strength from one to four grains of iodine with double the quantity of the iodide of potash to an ounce of water. It may be applied once or twice a day, or once every second or third day, as occasion may require. In some of the severer forms of this complaint, attended with considerable abrasion and ulceration, the diluted tincture may be used with great advantage.

Its curative powers are far greater than the nitrate of silver—which, in our hands, often seemed to exasperate the complaint—or any other remedy with which we are acquainted.—*Charleston Medical Journal and Review.*

